# Correlation of BMI with breast cancer subtype and tumour size

**DOI:** 10.3332/ecancer.2018.845

**Published:** 2018-06-26

**Authors:** K Govind Babu, Abhishek Anand, Kuntegowdanahalli C Lakshmaiah, Dasappa Lokanatha, Linu Abraham Jacob, MC Suresh Babu, Kadabur N Lokesh, Haleshappa A Rudresha, Lakkavalli K Rajeev, Smitha C Saldanha, GV Giri, Chethan R, Deepak Koppaka, Dipti Panwar, Rekha V Kumar

**Affiliations:** 1 Department of Medical Oncology, Kidwai Cancer Institute, Bengaluru 560029, India; 2 Department of Pathology, Kidwai Cancer Institute, Bengaluru 560029, India

**Keywords:** body mass index, breast cancer

## Abstract

**Background:**

Breast cancer is a heterogeneous disease which is divided broadly into luminal, HER2 and basal type based on molecular profiling. Increased body mass index (BMI) has been associated with the risk of developing breast cancer but the association based on molecular subtype remains conflicting.

**Methods:**

This was an observational study carried out over a period of 2 years. Nonmetastatic breast cancer patients were evaluated for the tumour subtype based on surrogate markers (ER, PR and HER2). The BMI of these patients was correlated with the tumour subtype and size.

**Results:**

We studied 476 patients with breast cancer with the median age of 46 years (range, 25–86) and 58% were premenopausal. The mean BMI of the cohort was 24.1, which was significantly higher in postmenopausal women (24.9 versus 23.6, *p* < 0.05). Overall, only 10% of patients were obese. The mean BMI in the luminal, HER2 and TNBC subtypes was 24.7, 22.4 and 23.9, respectively (*p* < 0.01). Also, the mean tumour size in luminal, HER2 and TNBC subtype was 4.02, 3.80 and 4.27 cm, respectively (*p* = 0.158).

**Conclusion:**

The average BMI was higher in patients with luminal subtype followed by TNBC and lowest for HER2 at the time of diagnosis. The mean tumour size was numerically higher for TNBC and lowest for HER2 subtype although the difference was not statistically significant. Larger studies may provide clarity of association between the BMI and tumour subtype.

## Introduction

Breast cancer is the most common cancer among females and is one of the commonest causes of cancer-related mortality worldwide [1]. In India, breast cancer is now the commonest cancer amongst women followed by carcinoma of the cervix with a rising incidence in young premenopausal women [2]. It is a well-known fact that breast cancer is a heterogeneous disease and has been divided into Luminal, HER2 enriched and basal-like subtype based on molecular profiling [3]. More recently, the genomic assays (oncotype DX, mammaprint, endopredict and Prosigna) are becoming a useful tool for defining the prognostic characteristics and in some cases for deciding the theraputic strategy. However, in routine clinical practice, breast cancer subtype is commonly approximated using the surrogate immunohistochemistry (IHC) markers, oestrogen receptor (ER), progesterone receptor (PR), human epidermal growth factor receptor 2(HER2) and Ki67 [4, 5]. An increased body mass index (BMI) has been found to be a risk factor for breast cancer in postmenopausal women. It is associated with more aggressive tumour biology and a poor prognosis [6, 7]. There are few studies that have found high BMI to be associated with a higher proliferation index, histological grade, a larger tumour size and a higher number of axillary node metastasis at the time of diagnosis. Some studies have also related a higher incidence of ER-positive breast cancer with BMI [8, 9]. However, it remains uncertain whether the same association is present in the Indian population which has different reproductive patterns compared to a western population and possibly a different subtype distribution as well. Moreover, the prevalence of obesity is not similar as compared to the western population [10]. The present study aims to find any association between BMI, tumour subtype and the breast cancer tumour size.

## Methods

This was an observational study of 476 breast cancer patients done at a tertiary cancer centre in India, from January 2016 to December 2017. Patients with a diagnosis of breast cancer and already having histopathology and IHC report of ER, PR, HER2 and Ki67 were included in the study. Patients underwent either modified radical mastectomy or breast conserving surgery depending on the stage. Those with *in-situ* carcinoma, nonductal pathology, bilateral breast cancer and metastatic disease were excluded from the study.

BMI was calculated using the Quetelet index using patient’s height and weight (BMI = weight in kg/height in meter^2^). The World Health Organization criteria were used to classify women based on BMI value into underweight and normal (BMI < 25); overweight (≥25 and <30) and obese (≥30) [11]. The tumour size measurement was based on the mammography and breast ultrasound. Imaging method was chosen for size criteria as a significant number of patients (32%) presented with locally advanced disease and were planned for neoadjuvant chemotherapy which could have underestimated the tumour size on surgical specimen. Tumour subtyping was done based on the surrogate IHC markers, ER, PR, HER2 and Ki67. Allred scoring was used for hormone receptor (HR) status and the American Society of Clinical Oncology/College of American Pathologists (ASCO/CAP) guidelines 2013 was used for reporting HER2 [12, 13]. For HR, 3+ or more was taken positive while 3+ on IHC was taken positive for HER2. Those equivocal for HER2 (2+ on IHC) was confirmed by doing fluorescent *in situ* hybridization. The primary tumour subtyping was done based on surrogate IHC markers according to the St Gallen criteria 2013 [14]. It subdivided breast tumours into luminal (A and B), HER2 over-expressing and basal (triple negative, TNBC) subtypes.

The BMI was correlated with the breast cancer subtype as well as the tumour size. For comparing the means of different variables, *t*-test and ANOVA were used. A *p* value of <0.05 was taken as significant. All analyses were done on SPSS, version 23.0 software.

## Results

We studied 476 patients of breast cancer who fulfilled the inclusion criteria over a period of 2 years. The median age at diagnosis was 46 years (range, 25–82). At the time of diagnosis, 278 (58.4%) patients were premenopausal whereas 198 (41.6%) were postmenopausal. The average age of menarche and first live birth was 13.7 and 23.4 years, respectively. Four patients were nulliparous while the rest of the cohort had an average of 2.4 children (range, 1–7). Breastfeeding was done by 468 women (98.3%). The mean BMI was 24.1 kg/m^2^ with postmenopausal women having significantly higher BMI than premenopausal women (24.9 versus 23.6; *p* = 0.02). Luminal type (53%; including luminal A and B) was the most common subtype followed by TNBC (31%) and HER2 enriched (16%). The patient characteristics have been given in Table 1. Most of the patients (58%) were in the BMI <25 group. Overweight (BMI 25–29.9) comprised 38% of the patients, whereas only 10% were obese (BMI ≥30) in the present study. The mean BMI in the luminal, HER2 and TNBC subtypes were 24.7, 22.4 and 23.9, respectively (*p* < 0.01). The relation of BMI and the breast tumour subtype has been given in Table 2. The tumour size according to the breast tumour subtype and BMI was also studied (Table 3). The mean tumour size in luminal, HER2 and TNBC subtype was 4.02, 3.80 and 4.27 cm, respectively (*p* = 0.158). The mean tumour size for BMI <25 was 3.99 cm; for BMI 25–29.9, 4.13 cm and for BMI ≥30 the mean size was 4.30 cm (*p* = 0.584).

## Discussion

We observed a higher BMI for luminal subtype at diagnosis followed by TNBC and HER2 subtype. Also, a higher BMI was associated with a larger tumour at presentation. TNBC subtype was associated with the maximum tumour size followed by luminal and HER2 in the present study. The exact relation of BMI and breast cancer remains conflicting even after numerous studies. Postmenopausal obesity has been a known risk factor for the occurrence of breast cancer [15]. However, there are studies in which higher BMI has been related to breast cancer risk in premenopausal women but not in postmenopausal women [16]. In a meta-analysis, the clinical importance of obesity on breast cancer risk was not seen [17]. There are studies showing a positive correlation of BMI with tumour size, stage and grade of the tumour [18–22]. BMI and its relation to the breast cancer subtype have also been studied, but with conflicting data. In a large retrospective analysis of 3,767 breast cancer patients, authors concluded that TNBC subtype had a significant association with premenopausal obesity [22]. Similar conclusions were made by other studies as well that analysed the BMI and its association with breast cancer subtype [23–25]. However, there are studies which have shown a more likely association of obesity with hormone positive breast cancer [26–29]. In the present study, HER2 (83%) and TNBC (60%) subtype have a higher proportion of women with BMI < 25 compared to the luminal subtype (49%). On the contrary, the proportion of patients with BMI ≥ 25 was higher in the luminal subtype (51%) compared to TNBC (40%) and HER2 (17%).

The clinical identification of the tumour is delayed in obese patients, which results in a larger tumour size at the time of diagnosis [30]. This has been shown in studies done by Daling *et al* [8], Loi *et al* [9], and Abrahamson *et al* [31]. In another large study by Biglia *et al* [19] on 2,148 women of breast cancer, there was a significant association of a higher BMI with larger tumour size at presentation. In this study, 45% of postmenopausal women with BMI > 25 had a tumour measuring more than 2 cm, compared to 33.4% in normal and 21% in underweight women. The mean tumour size in the present study for women with BMI < 25 was 3.99 cm; for BMI 25–29.9 was 4.13 cm and for BMI ≥ 30 was 4.30 cm. On tumour subtyping, the mean tumour diameter was maximum for TNBC (4.27 cm) and minimum for HER2 (3.80 cm).

In a study done by Castaneda *et al* [32], the TNBC subtype was more likely to present with a larger tumour when compared to luminal or HER2 subtype. They also found no significant difference in the T stage for the HER2 subtype. The present study shows that the aggressive subtype of breast cancer (HER2 and TNBC) was more likely to develop in nonoverweight women compared to the luminal subtype, which was more common in overweight and obese women. Also, the mean tumour diameter was maximum for TNBC and minimum for the HER2 subtype.

It is important to know the BMI, as many studies have shown a poor outcome associated with obese breast cancer patients [33–35]. Although this observation is fairly similar for luminal subtype, the outcomes for other subtypes remain conflicting. In a recent study using PAM-50 to define breast cancer subtype, the association of BMI with outcome was not dependent on the defined molecular subtype [36].

The biologic mechanism behind poor outcomes of breast cancer patients associated with obesity is multifactorial. Metabolic factors of obesity, e.g. insulin, glucose and adipokines like leptin, and inflammatory factors have been related to the poor outcomes. Among various factors, insulin plays an important role by binding to the insulin receptors expressed on breast cancer cells and activate signaling through PI3K/Akt and Ras/MAPK pathways, which finally leads increased tumour proliferation [37, 38].

Although obesity is associated with worse outcome, data pertaining to the effect of weight loss on outcomes after diagnosis is inconsistent. However, counselling for weight loss, lifestyle modification, physical activity and nutrition should be given to all breast cancer patients to maintain a healthy weight. The rationale for maintaining a healthy weight is for general health benefit as a high-level evidence of improving outcomes with weight control after diagnosis is still lacking.

## Conclusion

In conclusion, the present study shows that patients with luminal subtype breast cancer have the highest BMI at diagnosis followed by TNBC and HER2 subtypes. Higher BMI was associated with larger tumour at presentation and TNBC having the maximum tumour size followed by luminal and HER2. Large prospective studies are warranted for a better understanding of the association between the BMI and tumour characteristics, molecular subtype, as well as treatment outcomes.

## Figures and Tables

**Table 1.** Patient characteristics (*n* = 476).

Median age	46 (range, 25–82)
	
Premenopausal	278 (58%)
Postmenopausal	198 (42%)

Age of menarche (mean)	13.7 (range, 11–17)
Age at first live birth (mean)	23.4 (range, 17–34)
Number of children (mean)	2.4 (range, 0–7)
Nulliparous	4 (1%)
Breast feeding done	468 (98%)
	
BMI (kg/m^2^)	
Mean	24.1 (15.0-37.4, SD: 4.529)
Premenopausal	23.6 (15.0-37.4, SD: 4.507)
postmenopausal	24.9 (15.1-36.0, SD: 4.334)

Tumour subtype	
Luminal type	252 (53%)
HER2 enriched	77 (16%)
TNBC	147 (31%)

T status (tumour)	
T1	32 (6.7%)
T2	251 (52.7%)
T3	124 (26.1%)
T4	69 (14.5%)
N status (node)	
N0	136 (28.6%)
N+	340 (71.4%)

Stage	
I	17
II	212
III	247

**Table 2.** Number of patients according to BMI in each subtype of breast cancer.

Number of patients in each subgroup				
**BMI**	**Luminal (*n* = 252)**	**HER2 (*n* = 77)**	**TNBC (*n* = 147)**	**Total (*n* = 476)**
<25	125 (49%)	64 (83%)	88 (60%)	277 (58%)
25–<30	98 (39%)	11 (14%)	43 29%)	152 (38%)
≥30	29 (12%)	2 (3%)	16 (11%)	47 (10%)
Mean BMI	24.7 (15.0–37.4, SD:4.628)	22.4 (15.1–33.0, SD:3.711)	23.9 (17.5–37.5, SD:4.361)	24.1 (15.0–37.4, SD: 4.529)

**Table 3.** Mean tumour size in cms according to BMI and subtype.

BMI (kg/m^2^)	Luminal	HER2	TNBC	Total
<25	3.89 (1.5–11.0; SD: 1.886)	3.77 (1.6–10.0; SD: 1.912)	4.29 (2.0–9.0; SD: 1.484)	3.99 (1.5–11.0; SD: 1.781)
25–<30	4.1 (1.5–10.0; SD: 2.103)	3.94 (1.9–6.2; SD: 1.564)	4.15 (1.5–8.0; SD: 1.745)	4.13 (1.5–10.0; SD:1.963)
≥30	4.2 (1.5–7.4; SD: 1.554)	4.0 ( 3.5–4.5; SD: 0.707)	4.5 (2.5–7.5; SD: 1.811)	4.3 (1.5–7.5, SD: 1.605)
Mean (cm)	4.02 (1.5–11.0; SD:1.936)	3.80 (1.6–10.0; SD: 1.834)	4.27 (1.5–9.0; SD: 1.592)	4.06 (1.5–11.0; SD: 1.823)
